# Required temporal resolution for accurate thoracic aortic pulse wave velocity measurements by phase-contrast magnetic resonance imaging and comparison with clinical standard applanation tonometry

**DOI:** 10.1186/s12872-016-0292-5

**Published:** 2016-05-26

**Authors:** Karolina Dorniak, Einar Heiberg, Marcin Hellmann, Dorota Rawicz-Zegrzda, Maria Wesierska, Rafal Galaska, Agnieszka Sabisz, Edyta Szurowska, Maria Dudziak, Erik Hedström

**Affiliations:** Department of Noninvasive Cardiac Diagnostics, Medical University of Gdansk, Gdansk, Poland; Lund University, Skane University Hospital, Department of Clinical Sciences Lund, Clinical Physiology, Lund, Sweden; Department of Biomedical Engineering, Faculty of Engineering, Lund University, Lund, Sweden; 1st Department of Cardiology, Medical University of Gdansk, Gdansk, Poland; 2nd Department of Radiology, Medical University of Gdansk, Gdansk, Poland; Lund University, Skane University Hospital, Department of Clinical Sciences Lund, Diagnostic Radiology, Lund, Sweden

**Keywords:** Aorta, Pulse wave velocity, Temporal resolution, Magnetic resonance imaging, Phase contrast, Applanation tonometry

## Abstract

**Background:**

Pulse wave velocity (PWV) is a biomarker for arterial stiffness, clinically assessed by applanation tonometry (AT). Increased use of phase-contrast cardiac magnetic resonance (CMR) imaging allows for PWV assessment with minor routine protocol additions. The aims were to investigate the acquired temporal resolution needed for accurate and precise measurements of CMR-PWV, and develop a tool for CMR-PWV measurements.

**Methods:**

Computer phantoms were generated for PWV = 2–20 m/s based on human CMR-PWV data. The PWV measurements were performed in 13 healthy young subjects and 13 patients at risk for cardiovascular disease. The CMR-PWV was measured by through-plane phase-contrast CMR in the ascending aorta and at the diaphragm level. Centre-line aortic distance was determined between flow planes. The AT-PWV was assessed within 2 h after CMR. Three observers (CMR experience: 15, 4, and <1 year) determined CMR-PWV. The developed tool was based on the flow-curve foot transit time for PWV quantification.

**Results:**

Computer phantoms showed bias 0.27 ± 0.32 m/s for a temporal resolution of at least 30 ms. Intraobserver variability for CMR-PWV were: 0 ± 0.03 m/s (15 years), -0.04 ± 0.33 m/s (4 years), and -0.02 ± 0.30 m/s (<1 year). Interobserver variability for CMR-PWV was below 0.02 ± 0.38 m/s. The AT-PWV overestimated CMR-PWV by 1.1 ± 0.7 m/s in healthy young subjects and 1.6 ± 2.7 m/s in patients.

**Conclusions:**

An acquired temporal resolution of at least 30 ms should be used to obtain accurate and precise thoracic aortic phase-contrast CMR-PWV. A new freely available research tool was used to measure PWV in healthy young subjects and in patients, showing low intra- and interobserver variability also for less experienced CMR observers.

## Background

Aortic stiffness is related to progressive structural changes of the aortic wall with normal aging, [1] but also dependent on cardiovascular disease (CVD) risk factors such as hypertension independent of end-stage renal disease, glucose intolerance, diabetes, and obesity [2, 3]. Pulse wave velocity (PWV) can be used as a biomarker for aortic stiffness, and can predict CVD outcome beyond traditional risk factors [4]. Further, as arterial stiffness occurs earlier than luminal changes, [5] PWV has a potential as an early marker for atherosclerosis. The PWV is defined as the distance between two measurement planes divided by the difference in time between pulse waves at these measurement planes.

Cardiac magnetic resonance (CMR) imaging is increasingly used for cardiovascular assessment, and minor adjustments to a routine CMR protocol yields data for PWV. The CMR-PWV has been previously validated [6, 7] and is thus an attractive option for assessment of aortic stiffness, giving prognostic information at low extra cost.

Applanation tonometry (AT) is another method for assessing PWV, and this method is both generally available and well used in clinical routine as it is easy to apply [8]. The AT measures PWV on a more global arterial scale compared with CMR, between carotid and femoral arteries, whereas CMR usually is applied regionally in the aortic arch or thoracic aorta [9, 10]. The CMR-PWV measurements can however also be performed in a global fashion similar to AT-PWV covering the abdominal aorta or iliac arteries [1, 11]. Depending on the extent of aorta included in CMR-PWV measurements, the CMR-PWV values could be expected to not be interchangeable with AT-PWV, particularly important in follow-up studies.

All PWV methods are susceptible to sampling errors, CMR-PWV particularly so related to noise and temporal resolution, whereas the main limitation for AT-PWV is the estimated distance between flow curve measurement points. With the increasing availability of CMR, the paucity of available software to assess CMR-PWV is also viewed as a major limitation to the wider use of CMR-PWV [12].

The aims of this study were therefore to 1) investigate the acquired temporal resolution needed for accurate measurements of CMR-PWV; and 2) to provide a tool for CMR-PWV measurements and use this to compare CMR-PWV with the clinical standard AT-PWV, by experienced and less experienced CMR observers.

## Methods

### Study populations and protocol

The local Ethics Committee approved the study and all subjects provided written informed consent.

First, a set of simplistic computer phantoms was created in order to derive the adequate temporal resolution for accurate and precise quantification of CMR-PWV. The following acquisitions of CMR flow data in humans were performed using an acquired temporal resolution above this cut-off.

Irrespective of the PWV measurement method applied, the PWV tend to be lower and less dispersed in younger healthy individuals and higher and more scattered in older subjects, and also increase with presence of cardiovascular disease (CVD) risk factors. Therefore, PWV was obtained by both CMR and AT in younger healthy subjects and in older patients with CVD risk factors.

Healthy subjects (*n* = 13, median age 26 years, range 18 – 43 years, 5 women) were included in whom CMR was performed as part of routine workup due to positive family history or clinical findings raising suspicion of one of the following genetic/familial disorders: arrhythmogenic right ventricular cardiomyopathy, left ventricular non-compaction, Marfan syndrome or hypertrophic cardiomyopathy. In all included subjects the CMR results and other clinical examinations including genetic testing where appropriate were normal. The CMR was considered normal if all volumes, LV mass and thickness and regional and global function were within age and gender reference limits, and valvular pathology including regurgitation/stenosis was not present.

Consecutive patients (*n* = 13, median age 61 years, range 52 – 72 years, 7 women) were included with variable CVD risk profile including dyslipidaemia, hypertension, and history of coronary artery disease. Exclusion criteria were any acute cardiovascular condition such as myocarditis, acute coronary syndrome or overt heart failure within 3 months prior to inclusion, complex congenital heart disease, known familial or genetic cardiomyopathy or aortic stenosis or regurgitation.

Applanation tonometry was used as a clinical standard for comparison, and performed within 2 h after the CMR examination. The brachial arterial blood pressure was also measured in the supine position with a sphygmomanometer in all patients, in conjunction with AT.

### Computer phantoms

The velocity profiles and typical aortic centre-line distance between flow measurement planes were extracted from normal subject data with a heart rate of 60 beats per minute and 40 time frames, corresponding to a true temporal resolution of 25 ms. The velocity profiles were up-sampled with cubic interpolation to 10 000 time frames over a cardiac cycle, corresponding to a time resolution of 0.1 ms. Given a known PWV and the distance between flow measurement planes the temporal shift between flow curves was calculated. A set of such fixed temporal shifts was introduced to correspond to known PWV in the range of 2–20 m/s (in 10 steps of 2 m/s). After adding the temporal shift, the velocity profiles were down-sampled to between 20–40 time frames (21 steps per cardiac cycle), resulting in temporal resolutions of 25–50 ms. In the upsample/downsample process the highest temporal resolution thus was kept below the originally collected data based on 40 time frames, i.e. 25 ms. To ensure a mathematically correct down-sampling process optimal anti-aliasing filters were applied. The maximal percentage error between calculated PWV and true PWV was determined for each computer phantom by testing each combination of number of time frames and PWV. In total 210 (10 × 21) phantoms were evaluated. The determination of the cut-off for number of time frames to be used for adequate temporal resolution was based on visual inspection of identity plots and the quantitative bias between calculated and true PWV.

### CMR imaging

Patients were imaged in the supine position using either a 1.5 T MR scanner (Siemens Aera, Erlangen, Germany) with an 18-element phased array cardiac coil (*n* = 14) or a 3 T MR scanner (Philips Achieva 3.0 T TX, Eindhoven, the Netherlands) with a 32-channel InVivo cardiac coil (*n* = 12). Oblique sagittal slices covering the thoracic aorta (producing the so-called ‘candy-cane view’) and 1 phase-contrast image for aortic flow measurements at the level of the diaphragm were added to the routine protocol, which already included a phase-contrast flow acquisition in the ascending aorta. The oblique sagittal slices were acquired either with a HASTE (half fourier single shot turbo spin echo) or a 3D turbo spin-echo sequence. For quantitative flow measurements, a 2D phase-contrast gradient recalled echo (GRE) sequence with retrospective ECG-gating was used. Adequate velocity encoding (Venc) values were selected using a Venc-scout and set to between 150–250 cm/s for through plane flow quantification. Typical parameters for the quantitative flow phase-contrast GRE sequence were TR/TE = 20/3 ms, flip angle = 10/20°, 1.3 × 1.3 × 8 mm^3^. Fifty (Siemens) or 40 (Philips) velocity-encoded images were acquired per cardiac cycle, as determined after the computer phantom experiment. The typical segment size was approximately 20 ms for both scanners, ranging from 15 – 27.5 ms. The acquisition matrix in the phase encoding direction was adapted if necessary to meet a segment size < 30 ms.

### CMR image analysis

A new module for PWV was introduced in the software Segment (http://www.medviso.com) [13]. Aortic pulse wave travel distance was measured manually along the centre-line of the aorta in the oblique sagittal aorta images, between the acquired flow measurement planes in the ascending aorta and at the level of the diaphragm. For quantitative flow curves, automatic vessel segmentation was performed with manual corrections where needed. Delineations were performed in magnitude images and guided by phase-contrast images where appropriate.

**Fig. 1:**
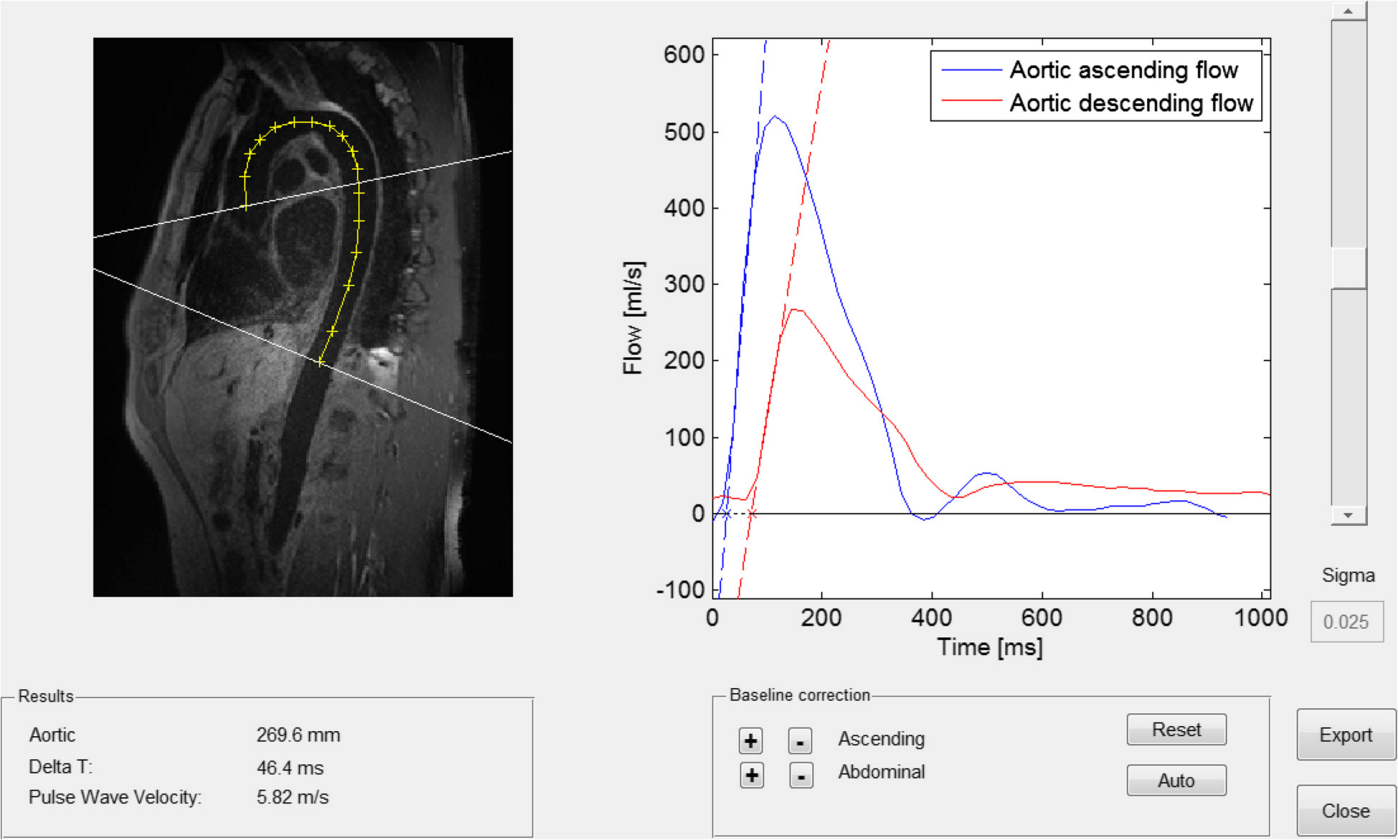
The graphical user interface for pulse wave velocity assessment. The oblique sagittal image of the aorta (left) shows the perpendicular flow measurement planes (white lines) in the ascending aorta and at the level of the diaphragm. The centre-line aortic distance between flow planes are shown as a yellow line with cross marks. The graph (right) shows the flow curves and respective upslope tangents with their baseline intersection points giving the time delay between flow curves (∆t; dashed part of the baseline tangent)

The flow curves from the ascending aorta and the aorta at the level of the diaphragm were superimposed and intersecting tangents between upslope and baseline were determined. The time-to-foot (TTF) approach was applied as this has been shown to be reliable for CMR-PWV [9]. In short, a regression line was fitted to the maximum upslope of each of the two flow curves representative of the ascending aorta and the aorta at the level of the diaphragm. The points at which these upslope tangents intersected the baseline tangents were labelled as the respective intersection points (Fig. 1). Prior to the upslope tangent fitting a Gaussian smoothing kernel was applied to the flow curves, corresponding to sigma = 0.62 ms (for a heart rate of 60 beats per minute; i.e. sigma value 0.025). The amount of smoothing was determined by visual inspection of the flow curves, and can be changed manually as needed. The time delay (∆t) between the intersection points was used as input for calculation of the CMR-PWV. The CMR-PWV was thus calculated by dividing the measured aortic centre-line distance between the ascending aorta and diaphragm level phase-encoded velocity flow planes by the ∆t between the 2 flow curves.

Due to concomitant or Eddy current background phase errors a baseline shift of the flow curves may be seen [14]. Built-in Eddy current compensation may solve this, but is also vendor-dependent. Therefore the PWV module also includes an option to adjust the individual flow curves to correct for this, both automatically and manually. The automatic method calculated the baseline as the mean flow rate during 62.5–87.5 % of the cardiac cycle. As this automatic correction may fail in cases with significant aortic regurgitation, a manual method for baseline correction was also implemented. Neither the manual nor the automatic method was however applied in the current study, as baseline shift was not seen.

Three observers blinded to other patient information and each other’s results performed independent CMR analyses. The observers had different experience in CMR (15 years, 4 years and less than 1 year). All observers also repeated their measurements more than 10 days later. The CMR-PWV data presented, except for comparisons between observers, are based on measurements by the most experienced observer.

### Applanation tonometry

Carotid-femoral PWV was measured noninvasively by AT according to recommended clinical standards [15]. Even though considered clinical standard today, as it is widely available, it is important to note that AT is not a gold standard. In the current study it is thus used as a comparison between non-invasive tools. Carotid and femoral pulse wave recordings were performed in supine subjects after 15 min of rest. Measurements were performed on the right common carotid artery [C] and the right femoral artery [F] using the widely available SphygmoCor device (AtCor Medical, Australia). Intravessel distance between measurement points was defined by approximation from body surface tape measurements and calculated as suprasternal notch to [F] - suprasternal notch to [C] [16].

The AT-PWV was calculated using commercial software (SphygmoCor Cardiovascular Management Suite; AtCor Medical, Australia) based on pulse transit time and the approximate intravessel distance between the two measurement points.

### Statistical analysis

Data were expressed as median (range) or mean ± SD. Statistical analyses were performed using Prism 6 (GraphPad Software, La Jolla, CA, USA). The observer variability was determined as bias ± SD and agreement was calculated according to Bland-Altman with 95 % limits of agreement. The Student’s two-tailed t-test was applied for testing of statistically significant differences in normally distributed data, whereas Fisher’s exact test was performed on categorical data, with *p* < 0.05 considered statistically significant.

## Results

Demographic and clinical characteristics are summarised in Table 1.

**Table 1:** Demographic and clinical characteristics of the study population

	Healthy young	Patients	*p*
Number of subjects	13	13	
Male/Female	8/5	6/7	0.70
Age (years)	26 (18 – 43)	61 (52 – 72)	<0.0001
BMI (kg/m^2^)	20 (17 – 24)	27 (22 – 35)	<0.0001
Systolic blood pressure (mmHg)	120 (100 – 140)	130 (110 – 160)	0.02
Diastolic blood pressure (mmHg)	80 (55 – 80)	80 (60 – 90)	0.17
History of smoking^a^	0	6	0.01
History of CAD	0	4	0.10
Statins	0	13	<0.0001
Antihypertensive drugs^b^	0	12	<0.0001
Diabetes^c^	0	2	0.48

^a^No current smoker. ^b^8 patients with ACE inhibitors, of which 1 with added diuretics, 1 with added calcium channel blockers, and 1 with added beta adrenolytics. Four patients with AT receptor blockers, of which 1 with added beta adrenolytics, 1 with added beta adrenolytics and diuretics, and 1 with added beta adrenolytics, diuretics and alpha adrenolytics. ^c^All patients without insulin dependent diabetes

### Computer phantoms

**Fig. 2:**
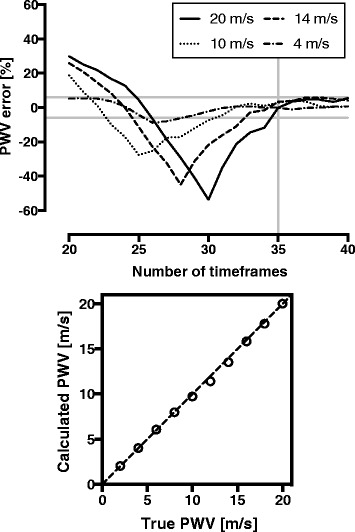
Median percentage PWV errors for number of time-frame samples for flow data from 210 computer phantoms. For clarity, 4 cases representative of different PWV are shown. All computer phantoms were however used for cut-off calculations. A plateau in the median PWV error is found from 35 time frames per cardiac cycle, corresponding to a temporal resolution of at least 30 ms at a heart rate of 60 beats per minute (top). The grey vertical line represents the cut-off 35 time frames, and the grey horizontal lines indicate the maximum PWV errors (±6 %) above the cut-off 35 time frames. The median error for 35 time frames was 0.27 ± 0.32 m/s between calculated and true PWV in phantoms, and is visualised in the identity plot (bottom). The dashed line indicates line of identity

The results of the computer phantoms are summarised in Table 2 and Fig. 2. The PWV error seems to increase with increasing number of time frames. The reason for this is that inadequate time resolution (number of time frames) generates smoothing, which prevents calculation of correct slopes and thus leads to incorrect PWV. For each true PWV there is a certain number of time frames where the error is maximal, and thereafter the error again decreases with increasing number of time frames. From 35 time frames per cardiac cycle, a plateau in the error of the PWV estimation is visible, corresponding to a required time resolution of at least 30 ms to accurately quantify PWV over a range of 2 – 20 m/s using phase-contrast CMR. The temporal resolution of at least 30 ms yielded a bias of 0.27 ± 0.32 m/s between calculated and true PWV in the computer phantoms. All human CMR flow data were thus acquired using a temporal resolution of at least 30 ms; i.e. set above 35 time frames also considering the individual cardiac frequency.

**Table 2:** The PWV median errors for different number of time frames/temporal resolution based on 210 computer phantoms

Time frames	Temporal resolution [ms]	Median error [m/s]	Median error [%]
20	50.0	2.36	21.3
21	47.6	1.51	17.2
22	45.5	0.72	10.4
23	43.5	0.16	9.1
24	41.7	-0.02	6.5
25	40.0	-0.39	4.4
26	38.5	-1.61	10.8
27	37.0	-2.74	18.1
28	35.7	-2.39	21.3
29	34.5	-1.83	16.2
30	33.3	-1.29	11.4
31	32.3	-0.76	6.7
32	31.3	-0.42	5.1
33	30.3	-0.20	2.6
34	29.4	-0.11	1.3
35	28.6	0.10	0.9
36	27.8	0.48	3.6
37	27.0	0.46	4.1
38	26.3	0.30	3.0
39	25.6	0.23	2.4
40	25.0	0.17	1.8

Errors are expressed as median errors for all velocities in the range of 2–20 m/s

For the Gaussian smoothing sigma value, a 50 % reduction in sigma from 0.68 ms to 0.34 ms resulted in a PWV change of -0.02 ± 0.12 m/s, indicating low impact on standard flow data. In images with a lower signal-to-noise ratio, however, a change in sigma may render more adequate fitting data.

### CMR

The CMR-PWV was feasible to perform in all subjects. As good signal-to-noise ratio was seen in all cases in the current study, sigma was not manually altered and thus sigma = 0.025 was used for all CMR-PWV analyses.

**Fig. 3:**
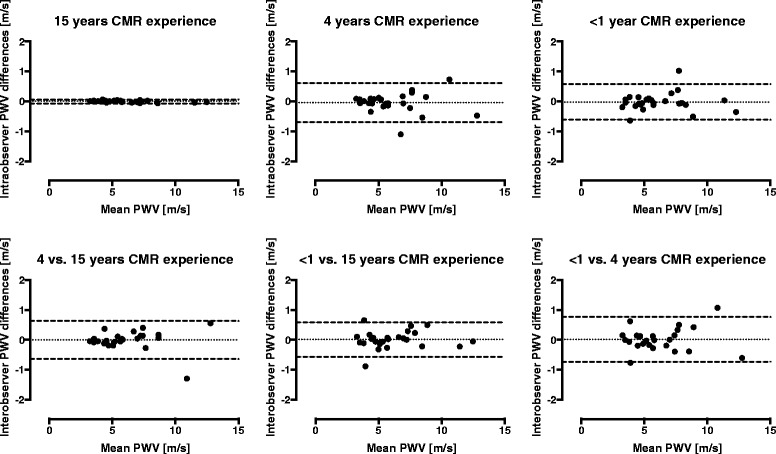
Bland-Altman plots showing intra- (top) and interobserver (bottom) CMR-PWV variability. Intraobserver variability for CMR-PWV was low for all 3 observers, 0 ± 0.03 m/s (15 years; left), -0.04 ± 0.33 m/s (4 years; middle), and -0.02 ± 0.30 m/s (<1 year; right), respectively. The interobserver variability was also generally low, for 4 vs. 15 years -0.01 ± 0.32 m/s (left), <1 vs. 15 years 0.01 ± 0.30 m/s (middle), and <1 vs. 4 years 0.02 ± 0.38 m/s (right). Dotted lines indicate mean differences and dashed lines represent 95 % limits of agreement

The CMR-PWV was statistically significantly higher in patients compared with younger healthy subjects (*p* < 0.0001; Table 3). Intraobserver variability for CMR-PWV for the 3 observers was 0 ± 0.03 m/s (15 years), -0.04 ± 0.33 m/s (4 years), and -0.02 ± 0.30 m/s (<1 year), respectively. The PWV interobserver variability between the 2 less experienced observers and the more experienced observer was for 4 vs. 15 years -0.01 ± 0.32 m/s and for <1 vs. 15 years 0.01 ± 0.30 m/s, and between the 2 less experienced observers PWV interobserver variability was 0.02 ± 0.38 m/s (Fig. 3). The observer variability can be related to aortic distance measurements between flow planes and flow measurements. Intraobserver variability for the aortic centre-line distance between flow planes was 0.2 ± 1.2 mm (15 years), -0.1 ± 4.8 mm (4 years), and -1.0 ± 5.9 mm (<1 year), respectively, with an average distance between flow planes of 250 ± 30 mm. Interobserver variability for aortic centre-line distance between flow planes, comparing the 2 less experienced observers and the more experienced observer, was for 4 vs. 15 years -1.2 ± 4.8 mm and for <1 vs. 15 years -1.9 ± 4.7 mm. Intraobserver variability for flow measurements, calculated as the time difference ∆t between flow curves was 0 ± 0.1 ms (15 years), 0.3 ± 1.7 ms (4 years) and 0.3 ± 2.5 ms (<1 year), respectively. Corresponding interobserver variability was for 4 vs. 15 years -0.2 ± 1.4 ms and for <1 vs. 15 years -0.3 ± 3.3 ms. Interobserver variability did not differ between 1.5 T and 3 T (all *p* > 0.12).

**Table 3:** PWV by AT and CMR

	AT-PWV (m/s)	CMR-PWV (m/s)	*p* (between methods)
Healthy young	5.6 ± 0.7	4.5 ± 0.8	<0.0001
Patients	9.0 ± 2.2	7.8 ± 2.2	0.07
*p* (between groups)	<0.0001	<0.0001

### Applanation tonometry

The AT-PWV values were, as for CMR-PWV, statistically significantly higher in patients than in younger healthy subjects (*p* < 0.0001; Table 3).

**Fig. 4:**
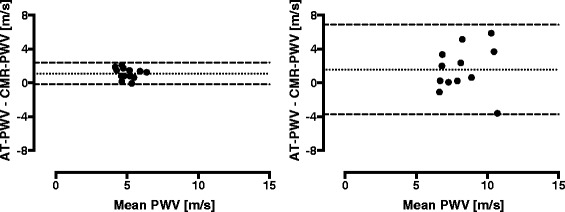
Bland-Altman plots showing AT-PWV vs. CMR-PWV. The AT-PWV overestimated CMR-PWV by 1.1 ± 0.7 m/s in younger healthy subjects (left) and by 1.6 ± 2.7 m/s in patients (right). Dotted lines indicate mean differences and dashed lines represent 95 % limits of agreement. Also note the PWV differences between younger healthy subjects and older patients where PWV is lower and less dispersed in healthy young subjects

The AT-PWV was statistically significantly higher than CMR-PWV for healthy young subjects (5.6 ± 0.7 vs. 4.5 ± 0.8; *p* < 0.0001). For patients a similar trend was found, however not statistically significant (9.0 ± 2.2 vs. 7.8 ± 2.2; *p* = 0.07) (Table 3; Fig. 4).

The bias and variability between AT-PWV and CMR-PWV were similar at 1.5 T and 3 T (1.5 T: 1.1 ± 2.4 m/s; 3 T: 1.6 ± 1.3 m/s; *p* = 0.56).

## Discussion

This study shows that an acquired temporal resolution of at least 30 ms is necessary to obtain accurate and precise quantitative flow data for CMR-PWV over the range 2–20 m/s, representative of PWV in the human aorta. Further, a new freely available tool for research was developed and used to measure CMR-PWV in healthy young subjects and in patients, showing low intra- and interobserver variability also for less experienced CMR observers.

The current study showed lower PWV in the younger healthy cohort compared with older patients at risk for CVD, as expected [1, 8]. The current study also showed slightly higher PWV for the healthy young cohort compared with data from an equivalent age group in a recent study including PWV in only the aortic arch (4 m/s), [10] but lower than a study also including parts of the abdominal aorta (5.6 m/s) [17]. This difference may be explained by that measurements for PWV are related to the elastic properties of the vessels, which vary along the arterial tree, with more elastic proximal segments and stiffer distal segments. In an invasive human study, pulse wave velocity increased from 5 m/s in the ascending aorta to 6 m/s in the abdominal aorta, and up to 9 m/s in the iliac and femoral arteries [18]. A recent CMR-PWV study however showed a different order of PWV, with highest PWV in the aortic arch (8–9 m/s), lower PWV in the iliofemoral segment (7–8 m/s), and lowest PWV in the thoracoabdominal segment (5–7 m/s) [11]. This difference is not explained and age groups are similar. Other CMR-PWV studies however also show approximately 4–6 m/s in the aortic arch, [1, 10] or approximately 6 m/s in the thoracic segment, [17] both comparable with the original invasive measurements by Latham et al. [18].

The CMR-PWV was determined with low intraobserver variability in the current study using the TTF approach, which has been previously shown to have high reproducibility [7, 9]. Further, also the less experienced CMR observers in the current study showed low interobserver variability versus the more experienced observer. A previous study showed similar interobserver variability between experienced observers (-0.1 ± 0.3 m/s), [9] which together with the current study indicates that CMR-PWV can be precisely performed also with less CMR experience.

The AT-PWV overestimated CMR-PWV by 1.1 ± 0.7 m/s in young healthy subjects and 1.6 ± 2.7 m/s in the older patients. This is a similar trend compared with previously published data where the corresponding reported differences were 0.43 m/s and 0.64 m/s [17]. Thus, PWV values are not interchangeable between AT and CMR. In AT-based measurements, age-related elongation or increased tortuosity of the aorta in disease may play a role, as this cannot be accounted for using standard surface tape estimates. Pure age-related elongation of the ascending aorta is considered to be of minor importance for AT-PWV, as this part of aorta is not included in distance measurements for calculation of AT-PWV [19]. When AT-PWV values are compared with CMR-PWV however also the ascending aorta is of importance but even more so the increased tortuosity of the abdominal aorta, which has impact on the vessel distance, not being accurate by surface tape estimates. In CMR-PWV accurate aortic centre-line distance measurements can be acquired in all cases, either by adequate angulation of a stack of oblique sagittal images covering the aorta, which can be combined into a 3D slab for proper distance measurements in software such as the one used in the current study, or directly assessed using 3D angiography techniques applying curved multiplanar reconstruction, with or without contrast agent.

The lack of availability of CMR-PWV software is currently viewed as a major limitation to the wider use of CMR-PWV [12]. The current study developed a new tool for measuring CMR-PWV, showing values corresponding to previous studies in comparable cohorts, and also a corresponding relation to AT-PWV. The 2D through-plane phase-contrast MRI method was chosen, as it is widely used in clinical CMR routine. This, together with the straightforward TTF method used in post-processing makes this approach attractive for clinical use. Other CMR-based PWV techniques are available, but are both more advanced and less elementary for clinical application [20, 21]. Further, the method applied in the current study has been shown to be the most reproducible in a recent comparison between transit time, cross-correlation and flow-area methods for CMR-PWV assessment [9].

### Limitations

The time of day, association with meals, sleep, caffeine and smoking may all impact PWV [15]. All these were corrected for in the current study except for the exact time of day the examination was performed. The differences related to time of day are however small and all subjects underwent AT within 2 h after CMR so potential changes are not considered to have impact on results. The distribution between 1.5 T and 3 T was not randomised but rather resulted from centre logistics. It is however unlikely that randomisation would significantly alter the results. The current study did not use invasive haemodynamics as a gold standard but rather compared two non-invasive methods, which may be considered a limitation. Invasive measures were however not necessary to show the readiness of PWV-CMR imaging and low variability using the proposed tool.

## Conclusions

Computer phantoms showed that an acquired temporal resolution of at least 30 ms (corresponding to 35 acquired time frames at a heart rate of 60 beats per minute) is necessary to obtain accurate and precise quantitative flow data for CMR-PWV over the range 2–20 m/s in the thoracic aorta. A new tool, freely available for research, was used to measure PWV in healthy young subjects and in patients showing low intra- and interobserver variability also for less experienced CMR observers.

## Abbreviations

AT: applanation tonometry
CVD: cardiovascular disease
CMR: cardiac magnetic resonance
ECG: electrocardiogram
GRE: gradient recalled echo
HASTE: half fourier single shot turbo spin echo
PWV: pulse wave velocity
TTF: time-to-foot
Venc: velocity encoding
